# Arctigenin from *Forsythia viridissima* Fruit Inhibits the Replication of Human Coronavirus

**DOI:** 10.3390/ijms25137363

**Published:** 2024-07-04

**Authors:** Jaeyeon So, Jang Hoon Kim, Siyun Lee, Chansoo Kim, Rackhyun Park, Junsoo Park

**Affiliations:** 1 Division of Biological Science and Technology, Yonsei University, Wonju 26493, Republic of Korea; jy_soh@yonsei.ac.kr (J.S.); lcy1230@yonsei.ac.kr (S.L.); ckstn201@yonsei.ac.kr (C.K.); 2 Department of Herbal Crop Research, National Institute of Horticultural & Herbal Science, RDA, Eumsung 27709, Republic of Korea; jhkim53@korea.kr; 3 Department of Life Science, Yong-In University, Yongin 17092, Republic of Korea; flowblue@yongin.ac.kr

**Keywords:** human coronavirus, forsythia viridissima, arctigenin, antiviral, natural compound

## Abstract

Coronavirus can cause various diseases, from mild symptoms to the recent severe COVID-19. The coronavirus RNA genome is frequently mutated due to its RNA nature, resulting in many pathogenic and drug-resistant variants. Therefore, many medicines should be prepared to respond to the various coronavirus variants. In this report, we demonstrated that *Forsythia viridissima* fruit ethanol extract (FVFE) effectively reduces coronavirus replication. We attempted to identify the active compounds and found that actigenin from FVFE effectively reduces human coronavirus replication. Arctigenin treatment can reduce coronavirus protein expression and coronavirus-induced cytotoxicity. These results collectively suggest that arctigenin is a potent natural compound that prevents coronavirus replication.

## 1. Introduction

Coronavirus causes various diseases from a mild common cold to severe and lethal COVID-19, and recently millions of people have died due to COVID-19 [1]. Before the COVID-19 era, the common cold was often caused by human coronavirus infection, and 15–30% of common cold cases are due to human coronavirus infection [2,3]. There are four popular human coronavirus strains (HCoV-229E, HCoV-NL63, HCoV-OC43, and HCoV-HKU1), and they can cause mild symptoms (upper respiratory tract infection). Because HCoV-OC43, SARS-CoV, MERS-CoV and SARS-CoV-2 belong to the beta coronavirus family, HCoV-OC43 is commonly used as a model system to study severe coronavirus-related diseases, including COVID-19 [4,5]. HCoV-OC43 is one of the seasonally-circulating endemic strains, and HCoV-OC43 infection causes mild colds [6]. For this reason, the experiments with HCoV-OC43 do not require strict laboratory conditions like a Biosafety level-3 (BSL-3). Therefore, HCoV-OC43 can be a beneficial model for studying severe coronaviruses like MERS and SARS-CoV-2.

Although vaccines and medicines for COVID-19 have been developed, there should be more methods to prevent and treat coronavirus diseases [7]. Coronavirus has an RNA genome and RNA viruses easily evolve into variants, which are often resistant to infection- or vaccine-elicited immunity [8]. Therefore, additional methods such as natural products should be developed to treat and prevent new coronavirus variants.

*Forsythia* species are distributed in Eurasia, including Korea, Japan, and China [9]. *Forsythia viridissima* fruit is widely used as a traditional herbal medicine to treat various diseases, such as fever and pain [10,11,12]. Recently, *Forsythia viridissima* extract was reported to have vasorelaxant, anti-inflammatory, antiasthmatic and neuroprotective effects [13,14,15,16]. Moreover, lignan components for *Forsythia viridissima* roots showed antiviral effects against coxsackievirus B3 and human rhinovirus 1B [17]. The fruit extract of *Forsythia viridissima* was used for these experiments, and the animal experiment with rats showed that oral administration of the fruit extract of *Forsythia viridissima* (single dose 5000 mg/kg) did not affect body weight, which suggests that the fruit extract of *Forsythia viridissima* is not toxic [10]. The main components of *Forsythia viridissima* extracts are lignans, phenylethanoid glycosides, flavonoids, and triterpenoids [14]. Previous analytical studies with *Forsythia viridissima* fruit extract demonstrated that *Forsythia viridissima* fruit extract contains three major constituents: arctiin, matairesinol, and arctigenin [15,16].

In this report, we attempted to find antiviral compounds that work against coronavirus and found that arctigenin from *Forsythia viridissima* fruit ethanol extract effectively reduces coronavirus replication.

## 2. Results

### 2.1. Forsythia viridissima Fruit Extract Treatment Reduces Coronavirus Replication

**Figure 1:**
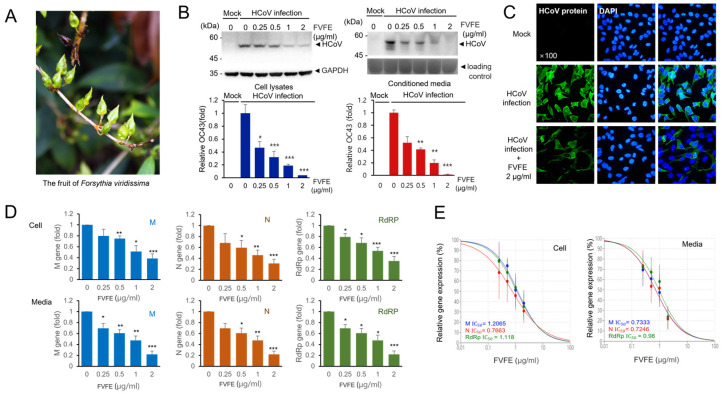
*Forsythia viridissima* fruit ethanol extract (FVFE) treatment reduces the expression level of HCoV protein and RNA. (**A**) The fruit image of *Forsythia viridissima*. (**B**) FVFE treatment decreases the level of HCoV protein expression. RD cells were infected with HCoV and treated with FVFE for 72 h. Cells and the conditioned media were collected and the expression level of coronavirus proteins was examined by Western blot with an anti-HCoV antibody (upper panels). Glyceradehyde-3-phosphate dehydrogenase (GAPDH) was used as a loading control. The level of HCoV proteins was quantified and depicted in the graph (lower panel). The error bars represent the standard error. Experiments were repeated at least three times. Control vs. FVFE treatment, * *p* < 0.05, ** *p* < 0.01, *** *p* < 0.001. (**C**) RD cells were infected with HCoV and treated with the indicated concentration of FVFE. The infected cells were fixed and stained with anti-HCoV antibody (green). The immune-stained cells were counterstained with DAPI (blue). (**D**) FVFE treatment decreases the level of HCoV RNA expression. Total RNA was isolated from the cell lysates and the conditioned media. Quantitative RT-PCR (qRT-PCR) was used to evaluate the relative level of HCoV RNA. Expression levels of membrane protein (M), nucleoprotein (N), and RNA-dependent RNA polymerase (RdRp) mRNA were evaluated. The error bars represent the standard error. Experiments were repeated at least three times. Control vs. FVFE treatment, * *p* < 0.05, ** *p* < 0.01, *** *p* < 0.001. (**E**) Half-inhibitory concentration (IC_50_) was determined from qRT-PCR analysis.

We searched for natural products that can reduce human coronavirus replication and used the human coronavirus OC43 (HCoV-OC43) strain to evaluate the antiviral effect of natural products by examining the expression level of human coronavirus proteins. During screening, we found that *Forsythia viridissima* fruit ethanol extract (FVFE) can reduce the expression of human coronavirus proteins. FVFE treatment reduced the expression of human coronavirus proteins in the cell lysates and the conditioned media in a dose-dependent manner (Figure 1B). Because the coronavirus proteins in the conditioned media are mainly derived from the newly-produced coronavirus particles, the reduction in coronavirus proteins in the conditioned media indicates the reduction in coronavirus replication. We also examined the expression of coronavirus proteins by immune-fluorescent staining and found that the expression of coronavirus proteins was reduced in the infected cells (Figure 1C). In addition, the number of infected cells was reduced by treatment with FVFE. These results indicate that FVFE treatment reduces coronavirus protein expression.

Next, we examined the RNA expression of human coronavirus in the cells and conditioned media using quantitative RT-PCR. We examined the RNA expression of human coronavirus membrane protein (M), nucleoprotein (N), and RNA-dependent RNA polymerase (RdRp) genes. We demonstrated that M, N, and RdRp RNA expressions were significantly reduced with FVFE treatment (Figure 1D). We calculated the half-inhibitory concentration (IC_50_) for FVFE, and IC_50_ was 0.7246–1.2065 µg/mL (Figure 1E). The RNA molecules in the conditioned media were from the human coronavirus RNA genome. Therefore, the reduction in RNA expression in the conditioned media indicates that FVFE treatment reduces the replication of the human coronavirus genome. These results collectively indicate that FVFE treatment can reduce coronavirus replication.

### 2.2. FVFE Treatment Reduces Coronavirus and Alleviates Cytopathic Effects

**Figure 2:**
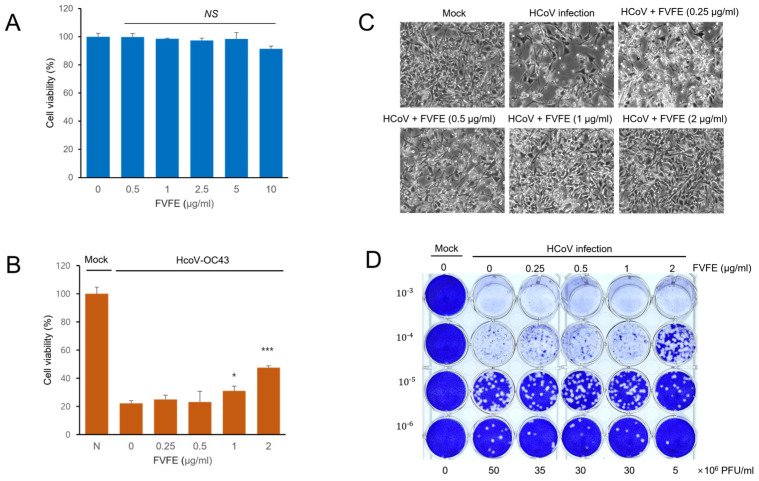
FVFE treatment reduces coronavirus-induced cytopathic effect in RD cells. (**A**) RD cells were treated with the indicated concentration of FVFE for 24 h, and cell viability was examined by MTT assay. The error bars represent the standard error. (**B**) RD cells were infected with human coronavirus (HCoV-OC43). Three days after infection, cells were treated with the indicated concentration of FVFE and cell viability was evaluated. Control vs. FVFE treatment, * *p* < 0.05, *** *p* < 0.001, NS, not significant. (**C**) Microscopic images of coronavirus-infected cells with FVFE treatment were obtained. (**D**) RD cells were infected with coronavirus and treated with the indicated concentration of FVFE. The conditioned media were used for plaque formation assay to evaluate the amount of coronavirus in the media. The number in the Y-axis indicates dilutions of conditioned media.

Because FVFE treatment reduced the replication of human coronavirus, we examined whether FVFE treatment alleviates coronavirus-induced cytotoxicity. Initially, we examined the cytotoxicity of FVFE by measuring cell viability upon treatment. FVFE did not decrease cell viability up to 5 µg/mL, and a slight decrease in cell viability was observed with the10 µg/mL treatment (Figure 2A). Next, we examined whether FVFE treatment alleviates coronavirus-induced cytotoxicity and whether FVFE treatment increases cell viability dose-dependently, suggesting that FVFE treatment reduces coronavirus-induced toxicity (Figure 2B,C). To enumerate the number of coronavirus particles after FVFE treatment, we performed a plaque formation assay. The conditioned media containing virus particles were collected and transferred to the new plates to form the plaque. FVFE treatment decreased the amount of virus-induced plaque formation in RD cells, and 2 µg/mL FVFE treatment reduced plaque formation by up to one-tenth (Figure 2D). These results indicate that FVFE treatment decreases the infectious coronavirus particles.

### 2.3. Arctigenin from FVFE Inhibits Coronavirus Replication

**Figure 3:**
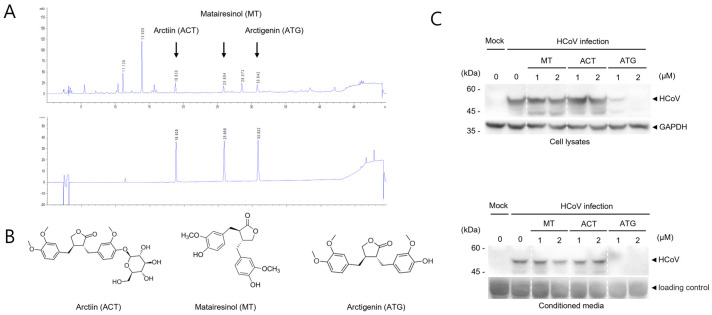
Active compounds in FVFE were determined by HPLC analysis. (**A**) FVFE was analyzed using the well-known main components of FVFE by HPLC analysis. The signals of ethanol extract of the dried fruits of *F. viridissima* (upper panel). The mixture signals of arctiin, matairesinol, and arctigenin (lower panel) (**B**) The structure of arctiin (ACT), matairesinol (MT), and arctigenin (ATG) are shown. (**C**) ATG is effective in reducing the level of coronavirus protein. RD cells were infected with HCoV and treated with ACT, MT, and ATG for 72 h. Cells and conditioned media were collected and analyzed using Western blot with an anti-OC43 antibody.

Because FVFE reduced the replication of human coronavirus, we attempted to find the active single compounds from FVFE. Arctiin (ACT), matairesinol (MT), and arctigenin (ATG) were known to be the components in the fruits of *F. viridissima* [15,16]. The retention times determined by HPLC of arctiin, matairesinol, and arctigenin were 18.8 min, 25.8 min, and 30.8 min, respectively. These signals were consistent with three main signals in the ethanol extracts, which were analyzed to contain three compounds (ACT, MT, and ATG) at 81.3 mg/g, 55.2 mg/g, and 79.7 mg/g, respectively (Figure 3A,B). Next, we examined the antiviral effect of ACT, MT, and ATG by Western blot. For this experiment, we used the single compounds purchased from the company as described in the methods. While MT and ACT treatment did not affect the expression of human coronavirus proteins, arctigenin (ATG) treatment decreased the expression of HCoV proteins (Figure 3C). These results indicate that arctigenin is a potent active compound of FVFE, which reduces coronavirus replication.

### 2.4. Arctigenin Treatment Shows the Inhibitory Effects to Human Coronavirus

**Figure 4:**
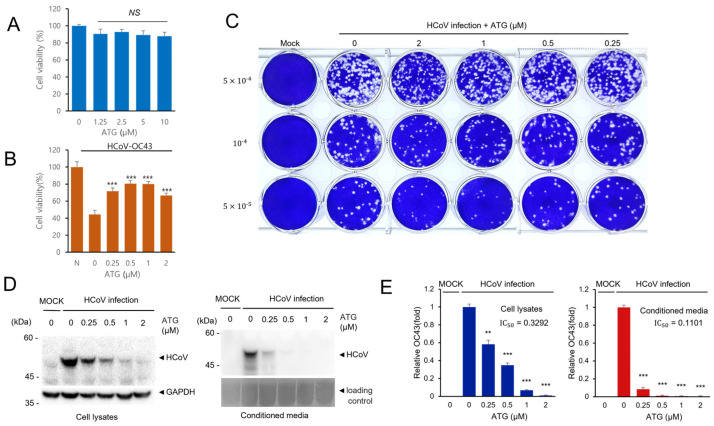
ATG treatment effectively reduces HCoV infection. (**A**) RD cells were treated with the indicated concentration of ATG, and cell viability was examined to determine the cytotoxicity of ATG. (**B**) ATG treatment alleviates HCoV-induced cytotoxicity. RD cells were infected with HCoV and treated with ATG. Cell viability was determined to examine the antiviral effect of ATG. Experiments were repeated at least three times. Control vs. ATG treatment, *** *p* < 0.001, NS, not significant. (**C**) ATG treatment reduces plaque formation. RD cells were infected with HCoV and treated with ATG. The plaque formation assay was performed to examine the antiviral effects of ATG. (**D**) ATG treatment decreases the level of HCoV protein expression. RD cells were infected with HCoV and treated with ATG for 72 h. Cells and the conditioned media were collected and the expression level of coronavirus proteins was examined by Western blot with an anti-HCoV antibody. (**E**) Bar graphs show the relative FECV protein expression level (HCoV protein/GAPDH). The error bars represent the standard error (*n* = 3). Control vs. ATG treatment, ** *p* < 0.01, *** *p* < 0.001.

Because arctigenin (ATG) treatment reduced coronavirus protein expression, we performed additional experiments to confirm the antiviral effects of arctigenin. First, we showed that arctigenin treatment had a minimal cytopathic effect up to 10 µM and that arctigenin treatment alleviates coronavirus-induced cytotoxicity (Figure 4A,B). Next, we examined the plaque formation of coronavirus with arctigenin treatment. Arctigenin treatment decreased the amount of plaque formation (Figure 4C). Moreover, arctigenin treatment decreased plaque size, indicating that arctigenin treatment decreased the cytotoxicity of coronavirus infection (Figure 4C). Finally, we confirmed the reduced expression of coronavirus protein after arctigenin treatment. RD cells were infected with HCoV-OC43 and treated with the indicated concentration of arctigenin. Arctigenin treatment decreased the coronavirus protein expression of cell lysates and media in a dose-dependent manner (Figure 4D,E). We also found that the coronavirus in conditioned media was strikingly decreased by arctigenin treatment, and these results suggest that arctigenin is a very potent compound that inhibits coronavirus replication.

## 3. Discussion

In this report, we attempted to find the active antiviral natural compounds from plants and found that arctigenin effectively reduces coronavirus replication. Initially, we performed functional screening to find plant extracts that can reduce coronavirus replication and found that *Forsythia viridissima* fruit ethanol extract (FVFE) effectively reduces coronavirus proteins. To demonstrate the inhibition of coronavirus replication, we measured the coronavirus protein and RNA in the cell lysate and media. The reduction in viral protein and RNA was statistically significant. We also evaluated the number of coronavirus particles produced in the presence of FVFE. The conditioned media were transferred to the new plate, and plaque formation was performed (Figure 2D). These results showed that FVFE reduces the number of infectious coronaviruses.

Because the plant extract contains many functional components, we attempted to find the functional components that inhibit coronavirus replication. Because HPLC analysis of *Forsythia viridissima* fruit extract was performed previously, we used the previous data for the FVFE main components (arctiin, matairesinol, and arctigenin) [15,16]. We confirmed that the current *Forsythia viridissima* fruit ethanol extract includes these main components using HPLC analysis. Next, we examined whether these single compounds effectively inhibit coronavirus replication and found that arctigenin effectively reduced coronavirus replication. Arctigenin effectively reduces coronavirus replication, and arctigenin treatment inhibits coronavirus-induced plaque formation. Because we examined three single compounds of *Forsythia viridissima* fruit extract, additional compounds can inhibit coronavirus replication.

Although many natural products were identified as adequate for bacterial or viral diseases in vitro, limited numbers of compounds were adequate to treat the disease in vivo. IC_50_ of many compounds from natural products is often high, often more than 1 µM (IC_50_). However, it does not exist easily in the body or blood up to 1 µM [18]. Arctigenin effectively reduces coronavirus replication at low concentrations (less than 0.25 µM). Therefore, arctigenin can be a potential compound for treating coronavirus. In addition, a higher concentration of arctigenin treatment (more than 5 µM) is reported to inhibit cancer cell proliferation, indicating that arctigenin can be cytotoxic at higher concentrations [19,20,21]. Further animal experiments should follow to demonstrate its activity in vivo.

When we compared the half-inhibitory concentration 50 (IC_50_) of arctigenin in the cell lysates and conditioned media, the IC_50_ of arctigenin in conditioned media was much lower than IC_50_ in cell lysates (Figure 4E). These data indicate that arctigenin effectively reduces the coronavirus in the conditioned media, and the virus production and release were inhibited by arctigenin treatment. We speculate that arctigenin possibly inhibits the process after coronavirus gene expression, such as the assembly of virus particles and the release of the virus from the cells. Therefore, the coronavirus particles in the conditioned media were strikingly reduced, although the viral proteins were expressed in the cell lysates.

Arctigenin is reported to have antiviral effects against influenza A virus, Chikungunya virus, Rhabdovirus and human immunodeficiency virus (HIV) [22,23,24,25,26]. Here, we showed that arctigenin is effective against coronavirus. Therefore, arctigenin can be used to prevent and treat multiple virus-related diseases. Previously, several in silico studies showed that arctigenin is a potent inhibitor of coronavirus papain-like protease, and we examined whether arctigenin inhibits coronavirus papain-like protease in vitro [27]. However, we failed to show the inhibitory effect of arctigenin on papain-like protease. Further study will be required to find the target of arctigenin, and the results will be beneficial in understanding its antiviral mechanism.

## 4. Materials and Methods

### 4.1. Material and Sample Preparation

The fruits of *Forsythia viridissima* Lindley were purchased from a traditional herbal medicine store in Nonsan City, Republic of Korea, in 2022, and authenticated by Ph.D. J.H. Kim. A voucher specimen (Sars2022-04) has been deposited at the herbarium of the Department of Herbal Crop Research, National Institute of Horticultural and Herbal Science, Republic of Korea. Arctigenin (#14913), arctiin (#15375), and matairesinol (#10005174) were purchased from the Cayman Chemical Company (Ann Arbor, MI, USA). The dried fruits of *F. viridissima* (4 g) were extracted three times with ethanol (40 mL). The extract solution was filtered and concentrated to achieve an ethanol extract (887.7 mg, yield 22.2%). The extract and compounds, filtered through 0.45 µm Millipore filters (Merck Millipore, Burlington, MA, USA), were prepared at 5 mg/mL and 0.5 mg/mL in methanol, respectively.

### 4.2. HPLC Analysis

Extract and compounds were analyzed using an Agilent 1260 HPLC system (Agilent Technologies, Santa Clara, CA, USA). A mobile phase was used for a distilled water gradient system (0.1% formic acid) and acetonitrile (0.1% formic acid). The gradient elution was 15% to 55%, 0 to 40 min; 55% to 100%, 40.1 to 43 min; 100% to 100%, 43.1 to 46 min; and 15% to 15%, 46.1 to 50 min. The column in chromatography was an Agilent eclipse plus 18 (5 µm, 4.6 × 250 mm). The detection wavelength, the column temperature, and the flow rate were 254 nm, 25 °C, and 1.0 mL/min, respectively.

### 4.3. Coronavirus Infection and Plaque Formation Assay

Rhabdomyosarcoma (RD) cells were maintained in MEM media (Welgene, Seoul, Republic of Korea) containing 10% FBS (Thermo Fisher Scientific, Waltham, MA, USA). RD cells were infected with human coronavirus (HCoV-OC43 strain, 10^7^ PFU/mL). RD cells were used for HCoV-OC43 infection because the virus yields were reported to be higher in RD cells [28]. A plaque formation assay was used to measure coronavirus titer. RD cells in a 12-well plate were infected with coronavirus and covered with the overlay medium containing 0.6% agarose. The infected cells were incubated for five days at 33 °C, fixed, and stained with crystal violet solution (1%). MTT assay measured cell proliferation as described previously [29]. Light microscopy was used to observe the cytopathic effect of coronavirus-infected cells using a Nikon TS100 inverted microscope (Nikon, Tokyo, Japan). HCoV-OC43 virus was obtained from ATCC (Rockville, MD, USA) and RD cells from Korean Cell Line Bank (Seoul, Republic of Korea). MTT reagent was purchased from Affymetrix (Cleveland, OH, USA).

### 4.4. Western Blotting and Immunofluorescence Assay

Western blotting was used to measure the coronavirus protein. Cells were collected and lysed with the lysis buffer (150 mM NaCl, 50 mM HEPES (pH7.5) and 1% NP40) containing a protease inhibitor cocktail (Roche, Basel, Switzerland). SDS-PAGE separated equal cell lysates, blotted into nitrocellulose membrane and probed with an anticoronavirus antibody (1:5000 dilution). Anti-HCoV-OC43 antibody was purchased from Sigma-Aldrich (Saint Louis, MO, USA). Western blot images were captured by Bio-Rad ChemiDoc Imaging Systems (Hercules, CA, USA). For immunofluorescence staining, cells were fixed with 4% paraformaldehyde and stained with an anticoronavirus antibody (1:1000 dilution) and Alexa-488 labeled secondary antibody (Thermo Fisher Scientific). Immunofluorescence images were captured by a Carl Zeiss LSM710 confocal microscope (Oberkochen, Germany). Crystal violet was purchased from Sigma, and agarose powder was purchased from Lonza (Rockland, ME, USA).

### 4.5. Quantitative RT-PCR for Coronavirus Genome

Quantitative RT-PCR (qRT-PCR) was used to measure the level of coronavirus RNA. Cells and the conditioned media were collected, and RNA was extracted using TRIZOL reagent (Thermo Fisher Scientific) according to the manufacturer’s protocol. An equal amount of RNA was used for reverse transcription for cDNA production, and qRT-PCR was used to measure coronavirus RNA [30]. StepOnePlus Real-Time PCR system (Thermo Fisher Scientific) was used for qRT-PCR. Coronavirus membrane protein (M) gene was amplified using the forward primer 5′-ACG GTC ACA ATA ATA CGC GGT-3′ and reverse primer 5′-GGG TTG ATG GCA GTC GGT AA-3′. Coronavirus nucleoprotein (N) gene was amplified using the forward primer 5′-AGG ATG CCA CCA AAC CTC AG-3′ and reverse primer 5′-TGG GGA ACT GTG GGT CAC TA-3′. Coronavirus RNA-dependent RNA polymerase (RdRp) gene was amplified using the forward primer 5′-GAG TGT AGA TGC CCG TCT CG-3′ and reverse primer 5′-TGT GGC ACA CGA CTA CCT TC-3′. PCR primers were synthesized in Macrogen (Seoul, Republic of Korea). cDNA synthesis kit was purchased from Enzynomics (Seoul, Republic of Korea).

### 4.6. Statistical Analysis

For cell proliferation, coronavirus protein quantitation, and qRT-PCR results, the statistical significance was evaluated with a 2-tailed Student’s *t*-test using Excel Software 2021 (Microsoft, Redmond, WA, USA). The results are considered statistically significant when a *p*-value is lower than 0.05. An IC_50_ calculator was used to calculate the half-inhibitory concentration (https://www.aatbio.com/tools/ic50-calculator (accessed on 1 April 2024)).

## 5. Conclusions

Because coronavirus produces many variants, including drug-resistant viruses, many medicines should be prepared for these variants. This study found that *Forsythia viridissima* fruit ethanol extract (FVFE) effectively reduces coronavirus replication. FVFE treatment decreases coronavirus mRNA and protein and also reduces plaque formation. Arctigenin, arctiin, and matairesinol are reported to be significant components of FVFE, and we demonstrated that arctigenin effectively reduces the replication of coronavirus. Therefore, arctigenin is potentially helpful in treating coronavirus-related diseases.

## Data Availability

Data are contained within the article.
